# A novel Huntington's disease mouse model to assess the role of neuroinflammation on disease progression and to develop human cell therapies

**DOI:** 10.1002/sctm.20-0431

**Published:** 2021-03-12

**Authors:** Heather Dahlenburg, David Cameron, Sheng Yang, Angelica Bachman, Kari Pollock, Whitney Cary, Missy Pham, Kyle Hendrix, Jeannine White, Haley Nelson, Peter Deng, Joseph S. Anderson, Kyle Fink, Jan Nolta

**Affiliations:** ^1^ Stem Cell Program and Institute for Regenerative Cures University of California Davis Health Sacramento California USA; ^2^ Department of Neurology University of California Davis Health Sacramento California USA; ^3^ Department of Internal Medicine University of California Davis Health Sacramento California USA

**Keywords:** humanization, Huntington's disease, mouse model, neuroinflammation, stem cells

## Abstract

Huntington's disease (HD) is a fatal autosomal‐dominant neurodegenerative disease caused by a trinucleotide CAG repeat expansion of the huntingtin gene (*HTT*) that affects 1 in every 10 000 individuals in the United States. Our lab developed a novel immune deficient HD mouse strain, the YACNSG, from a commonly used line, the YAC128 mouse, to enable transplantation studies using engineered human cells in addition to studying the impact of the immune system on disease progression. The primary goal of this project was to characterize this novel immune deQficient HD mouse model, using behavioral assays and histology to compare this new model to the immune competent YAC128 and immune deficient mice that had engraftment of a human immune system. Flow cytometry was used to confirm that the YACNSG strain lacked immune cells, and in vivo imaging was used to assess human mesenchymal stem/stromal cell (MSC) retention compared with a commonly used immune deficient line, the NSG mouse. We found that YACNSG were able to retain human MSCs longer than the immune competent YAC128 mice. We performed behavioral assessments starting at 4 months of age and continued testing monthly until 12 months on the accelerod and in the open field. At 12 months, brains were isolated and evaluated using immunohistochemistry for striatal volume. Results from these studies suggest that the novel immune deficient YACNSG strain of mice could provide a good model for human stem‐cell based therapies and that the immune system appears to play an important role in the pathology of HD.

## Graphical abstract

To create the novel HD strain, the YAC128 was backcrossed with NSG mice to produce an immune deficient HD mouse model.

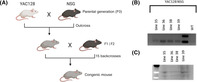

**Significance statement**

The present manuscript describes a novel, immunodeficient mouse model of Huntington's disease. This mouse strain was extensively characterized and is valuable to the research community due to the ability of not rejecting human cell products. The study also describes mice that had their immune system reconstituted with a human immune system. These studies further delineate the role of the immune system in neurodegenerative diseases.

## 1 INTRODUCTION

Huntington's disease (HD) is a fatal autosomal‐dominant neurodegenerative disease that currently has no cure. It is characterized by the progressive onset of chorea, behavioral and psychiatric changes, and cognitive decline.^1^, ^2^ The loss of efferent medium spiny neurons (MSN) in the striatum, located in the subcortical basal ganglia of the forebrain, results in a measurable decline in striatal volume, followed by general whole brain atrophy in HD patients.^3^ HD is caused by a trinucleotide CAG repeat expansion in exon 1 of the huntingtin (*HTT*) gene, located on chromosome 4.^4^ This leads to a mutated huntingtin (mHTT) protein which has an elongated stretch of glutamine at the N‐terminus, resulting in a misfolded protein.^4^ The misfolded HTT protein can fold and stick together in clumped, rigid aggregates. Once aggregates form, they tend to accumulate in inclusion bodies where they are sequestered.^3^, ^5^ Evidence suggests that aggregates interfere with normal cellular function, including axonal transport between the cell body and the synaptic terminal,^6^ leading to disease progression.

Neuroinflammation was originally used to describe events such as ischemic stroke, traumatic brain injury, multiple sclerosis, or viral/bacterial infections that allowed infiltration of peripheral immune cells into the brain. Currently, the study of neuroinflammation has expanded to include neurodegenerative diseases such as Huntington's disease that do not attract inflammatory cells from the periphery but are characterized by cellular and molecular features of inflammation such as alterations of cytokine expression and microglia activation.^7^ In postmortem HD brains, accumulation of reactive microglia and astrocytes has been observed in addition to PET/MR imaging, suggesting that neuroinflammation begins to accumulate in the basal ganglia of HD patients and could serve as a valuable biomarker and target for clinical interventions.^7^, ^8^ The role of neuroinflammation in HD is still a matter of debate. Neuroinflammation may play an important role early in disease progression as a mechanism to promote clearance of cell debris and protein aggregates.^9^ However, as the disease progresses neuroinflammatory mechanisms may play a role in neuronal death which in turn would activate further neuroinflammation resulting in a vicious cycle of neurodegeneration and inflammation.^7^, ^9^

An important aspect of HD research is the development and use of transgenic mouse models that express the human mHTT protein. The HD transgenic mouse model termed YAC128 has been characterized as an accurate replication of adult onset HD. It recapitulates the slow decline in motor and behavioral function and the progressive striatal loss and neuropathology that are seen in human HD.^10^ The mutation in the YAC128 mice was created by microinjection into pronuclei.^10^ The YAC128 mice have a human HTT gene with 128 CAG repeats on a yeast artificial chromosome, which is an autonomous chromosome.^10^ The YAC128 mouse line expresses the full length human mutated *HTT* gene as well as the promoter, introns, upstream, and downstream regulatory elements.^10^ YAC128 mice begin displaying HD symptoms at 3 months of age and are considered presymptomatic.^10^ At this stage they display a hypokinetic phenotype, followed by motor deficits on the rotarod by 6 months of age and progression to hypokinesis by 12 months.^10^ The symptoms indicate loss of muscle movement over time, similar to that which is seen in human HD patients. These behavioral changes correlate to neuronal loss. Striatal atrophy is evident at 9 months of age with cortical atrophy by 12 months of age.^10^

The YAC128 strain is an excellent model for HD. However, this mouse model must be immune suppressed in order to study human cell‐based therapies. Past studies with the YAC128 mouse model have used immune‐suppression drugs in order to reduce the immune response in mice, allowing human cells to survive longer in vivo to test potential therapies. A previous study from our lab showed that human mesenchymal stem cells (hMSC) could only survive in vivo for 7 days without immune‐suppression drugs, but up to 28 days with them in the YAC128 strain.^11^ hMSC have gained much interest in the field for their ability to modulate inflammation with several clinical trials aimed at autoimmunity.^12^, ^13^ However, these immune‐suppression drugs have toxic effects on the mice over time^14^, ^15^, ^16^, ^17^ and do not result in a completely immune deficient animal, making the drugs inefficient for studying human stem cell based therapies for HD.

Here, we generated and characterized an immune deficient and xeno‐tolerant mouse model of Huntington's disease with the human mutated *HTT* gene by cross‐breeding the NOD.Cg‐Prkdc^scid^Il2rγ^tm1Wgl^/SzJ (NSG) mouse,^18^, ^19^ a well‐known immune deficient model, with YAC128 mice. This resulted in a novel immune deficient HD mouse model, referred to as YACNSG. In addition, we examined the role of the human immune system in contributing to the pathogenesis of HD by humanizing the YACNSG mice. It is hypothesized that the accumulation of activated microglia in response to mHTT protein aggregates plays a role in disease progression. The development of these mouse strains allows for the study of human stem cell products without the need for immunosuppression, as well as the study of the role of the immune system in the progression of disease in murine models of Huntington's disease.

## 2 METHODS

All mouse studies were performed under UC Davis IACUC approval. Umbilical cords designated for research were acquired from the California Umbilical Cord Blood Collection Program under IRB approval.

### 2.1 Strain development

To generate the YAC128/NSG strain we performed 15 backcrosses to introduce the full length human HD transgene of the FVB‐Tg(YAC128)53Hay/J (YAC128) mouse model of HD onto the xenotolerant NOD.Cg‐PrkdcscidIl2rγtm1Wgl/SzJ (NSG).

### 2.2 DNA/RNA/protein analysis of YACNSG strain

A 4 mm section of brain tissue was isolated for DNA/RNA analysis. Genotyping was performed as previously described using published primers.^20^ Genotyping primers were designed from the Jackson Laboratory genotyping protocol. Primers used were as follows: Transgene forward primer CCG CTC AGG TTC TGC TTT TA, transgene reverse primer TGG AAG GAC TTG AGG GAC TC. Western blot was performed using MAB2166 (Millipore).

### 2.3 Flow cytometry of YACNSG strain

To determine the presence of immune cells in the YACNSG strain, red‐blood‐cell‐lysed peripheral blood, spleen, and bone marrow were collected and labeled with antibodies specific for mouse immune cell markers and analyzed by flow cytometry. All immune cells were labeled with BUV395‐conjugated CD45 (clone 30F11). T cells were labeled with an APC‐Cy7‐conjugated CD3e (clone 145‐2C11), BUV737‐conjugated CD4 antibody (clone RM4‐5), and PE‐CY7‐conjugated CD8 antibody (clone 53‐6.7). B cells were labeled with an APC‐conjugated CD45R antibody (clone RA36B2) and a BB515‐conjugated CD19 antibody (clone 1D3) (BD Biosciences). NK cells were labeled with a PE‐conjugated CD335 antibody (clone 29A1.4) and a BV421‐conjugated CD49B antibody (clone DX5) (BD Biosciences). Compensation was completed using BD positive/negative compensation beads and an ArC Reactive Compensation bead kit. Viability was assessed with the ZOMBIE Aqua Fixable Viability Kit (Biolegend). Flow cytometry was performed on a BD Fortessa and analyzed with FloJo software (Beckman Coulter).

### 2.4 MSC isolation, transduction, and culture

Whole bone marrow was purchased commercially (Lonza, Walkersville, Maryland) and mesenchymal stem cells (MSCs) were isolated using a Ficoll‐Paque density gradient as previously described.^2^ Isolated cells were plated and expanded in T225 culture flasks in Complete Culture Medium (CCM) composed of Modified Essential Medium Eagle—Alpha (aMEM) (HyClone, Marlborough, Massachusetts) with added supplements of 10% fetal bovine serum (Atlanta Biologicals, Flowery Branch, California), 1% l‐glutamine (HyClone), and 1% penicillin/streptomycin (HyClone). Non‐adherent cells were removed after 24 hours and MSCs were expanded with fresh media. Expanded cells were then transduced with the pCCLc‐MNDU3‐LUC‐PGK‐EGFP‐WPRE lentiviral vector containing the luciferase gene following previously published protocol.^11^ Briefly, Lenti‐X 293 cells (Clonetech, Mountain View, California) were transfected with the transfer and packaging plasmids, and the virus was harvested after 52 hours. The concentrated virus was then added to the MSC culture at a multiplicity of infection (MOI) of 60 in the presence of 10 mg/mL protamine sulfate (MP Biomedical, Santa Ana, California). After 24 hours the media was replaced with fresh CCM and the cells were expanded following normal culture protocol at 37°C and 5% CO_2_. Forty‐eight hours prior to implantation, cells were moved to a 5% CO_2_/1% O_2_ incubator for hypoxic preconditioning.^21^, ^22^ On the day of injection, the cells were lifted by trypsinization (HyClone) and were resuspended in Normosol‐R (Hospira, Inc, Lake Forest, Illinois) at a final concentration of 50 000 cells/mL.

### 2.5 MSC transplantation for IVIS imaging study

Animals used in this study were generated from the breeding colony at the UC Davis Institute for Regenerative Cures (Sacramento, California). Prior to surgery, mice were anesthetized using isoflurane (2‐3% in oxygen) and placed in a stereotaxic frame. The hair was removed using Nair and skin was cleaned three times with alternating betadine solution and alcohol. A small incision (<1 cm) was made on the scalp and a 1 mm burr hole was drilled into the skull at +0.5 mm AP, +1.7 mm ML relative to bregma. Cells were injected into the striatum at a depth of −2.5 mm using a Hamilton syringe and automated microsyringe pump. The cells were injected at a rate of 0.5 μL/min with a total volume of 5 μL. Five minutes post injection, the needle was withdrawn and the incision sutured using 6‐0 silk suture. Carprofen was administered by subcutaneous injection (5 mg/kg) at the time of surgery and again the following day.

### 2.6 In vivo imaging

Cell survival of the transplanted MSC was evaluated using the in vivo imaging system (IVIS) spectrum instrument (Perkin Elmer, Waltham, Massachusetts). Mice were given an intraperitoneal (IP) injection of luciferin (Xenolight D‐luciferin K+ Salt, Perkin Elmer) at 3 mg/mouse 15 minutes prior to imaging. Animals were anesthetized with isoflurane 10 minutes prior to imaging. Imaging was done on days 2, 4, 7, 14, and then weekly until week 6 post‐op. Animals were euthanized by CO_2_ asphyxiation upon completion of the study.

### 2.7 Humanization of YACNSG mice

Humanization of YACNSG mice followed established protocols.^23^, ^24^, ^25^ Briefly, human CD34+ hematopoietic stem and progenitor cells (HSPC) were isolated from umbilical cord blood obtained from the UC Davis Umbilical Cord Collection Program by Ficoll‐Paque (GE Healthcare, Logan, Utah) density gradient. They were further purified by CD34 magnetic bead column separation (Miltenyi Biotec, Auburn, California). Mice were sublethally irradiated with 125 rads 2‐5 days after birth. Irradiated mice were intrahepatically injected with 2‐5 × 10^5^ purified CD34+ cells with up to 30 μL of volume using an insulin syringe. Twelve weeks postimplantation, blood was collected from the tail vein to test for human cell engraftment. Flow cytometry was conducted to identify the level of engraftment using a PE‐CY7‐conjugated anti‐human CD45 antibody (BD Biosciences, Clone‐HI30), a pan‐leukocyte marker. Flow cytometry was performed using a Beckman Coulter FC‐500. Transgenic and wildtype mice were chosen for the behavior study by matching engraftment levels. Ten male and 13 female transgenic, along with 10 male and 15 female wildtype mice, were used for behavioral testing.

### 2.8 Functional assessment

For this study, mice were used from an established breeding colony at the UC Davis Institute for Regenerative Cures. YACNSG and YACNSG‐humanized mice were tested once a month with YAC128 transgenic and wildtype littermate controls beginning at 4 months of age. All functional and histological assessments were performed blinded to both the genotype and engraftment status. At the conclusion of all data collection the experimenters were unblinded to perform data analysis.

### 2.9 Rotarod

Mice were initially trained on the rotarod for 3 days; three trials/day at a fixed speed of 5 rpm for 120 seconds. Mice that fell off the rod were placed back on for the duration of each trial. The final day of training was completed 3 days prior to the first testing day. Mice were tested on the accelerating rotarod (4‐40 rpm over 360 seconds) three trials each testing day with a resting period between each trial. Total amount of time spent on the rod was recorded by Rotamex system software (Columbus Instruments).

### 2.10 Open field

Mice were placed in an open arena for 10 minutes and monitored by Omnitech Electronics, Inc. Fusion system software version 4. Data was collected on total distance traveled, stereotypic episodes, time spent in the center, rest time, and rearing events. A stereotypic episode occurs when the mouse does a repetitive movement, most commonly grooming. A rearing event is when the mouse puts weight on its hind legs and lifts the forelimbs.

### 2.11 Histology

At the conclusion of behavior testing, mice were euthanized by CO_2_ asphyxiation followed by bilateral thoracotomy. Mice were perfused with 10 mL PBS (HyClone) followed by 10 mL formalin (Fisher Healthcare, Pittsburg, Pennsylvania). Brains were harvested, fixed in formalin for 24 hours, then transferred to 30% sucrose (Fisher Chemical, Fair Lawn, New Jersey) for 24‐48 hours at 4°C. Brains were then frozen for 3 minutes in a dry ice/100% isopropanol bath at −77°C and then stored at −80°C until further processing.

### 2.12 Immunohistochemistry analysis

Brains were serially sectioned at 30 μm using a Cryotome FSE and placed into a 12 well plate filled with PBS with about 14 sections per well. The wells were put in 10% SEA BLOCK (Thermo) for an hour on an orbital shaker at 60 rpm. Sections were moved into primary solution: mouse anti‐rabbit MAP2 (1:1000, Abcam) and rabbit anti‐mouse DARPP32 (1:1000, Abcam) and placed on a 4°C orbital shaker overnight. Sections were then washed with PBS three times and placed into secondary antibody stain consisting of 5% SEA Block, AlexaFluor 488 anti‐mouse (1:1000, LifeTech), and AlexFluor 647 anti‐rabbit (1:1000, LifeTech) on a 4°C orbital shaker for an hour. After incubation, the wells were spiked with Hoescht (1:1000) for 5‐10 minutes on an orbital shaker, then transferred to phosphate buffered saline plus 1% Triton‐X (PBST) and washed three times. To utilize the autodetection software on the AxioScan, sections were immersed in 0.01% Sudan Black in 70% EtOH, gently agitated for 1 minute and then allowed to rest for 1 minute before being transferred into fresh PBS. The sections were then mounted onto microscope slides (Thermo Fisher Scientific) sequentially from the beginning to the end of the striatum and covered with a coverslip using Fluoromount (Sigma). Whole brain sections were then scanned and stitched together at 20× using a Zeiss AxioScan.

### 2.13 Statistical analysis

SPSS v26 was used to perform all statistical analyses. Behavioral data was analyzed using a repeated measures analysis of variance (ANOVA) to measure changes between genotypes across timepoints. When appropriate, an LSD post hoc analysis was completed. One‐way ANOVA was performed at the 12 month timepoint for striatal atrophy followed by an LSD post hoc analysis when appropriate.

## 3 SUPPLEMENTAL METHODS

### 3.1 Flow cytometry

Mice were euthanized CO_2_ and blood, spleen, and bone marrow were isolated. Blood (approximately 1 mL) was collected from the body cavity of the mouse using vacuum capillary tubes and incubated with 1X RBC lysis buffer (eBioscience) for 10 minutes. Following lysis, the sample was centrifuged at 3000 rpm for 3 minutes. The supernatant was removed, and the remaining pellet was washed with 1 mL PBS. Resuspended cell pellets were divided evenly between several tubes and centrifuged at 3000 rpm for 3 minutes. Supernatant was removed and the sample was resuspended in 100 μL PBS and placed on ice until ready to label with antibodies.

The spleen was removed from the animal and placed onto a 70 μM strainer cap atop a 50 mL conical tube. Using a 3 mL syringe, the spleen was “popped” open and washed with 2 mL PBS. The spleen was repeatedly minced and washed with 2 mL PBS for a total of 3 times. The cell suspension was transferred to a 15 mL conical and centrifuged at 2000 rpm for 4 minutes. The cell pellet was resuspended in 2.5 mL PBS and cell clumps were removed. Sample was aliquoted and stored on ice until ready to stain.

Bone marrow was collected from the leg bones of mice into PBS. Briefly, the femur and tibia were removed from the mouse the head of either bone was cut and a syringe with PBS was inserted into the end of the bone. The bone marrow was then pushed through the bone and collected in PBS. The cell suspension was passed through a 70 μM cell strainer cap atop a 50 mL conical tube and transferred into a 15 mL conical before centrifuging for 4 minutes at 2000 rpm. After removing the supernatant, the pellet was resuspended in PBS up to 300 μL volume. The sample was aliquoted and stored on ice until ready to stain.

## 4 RESULTS

**FIGURE 1:**
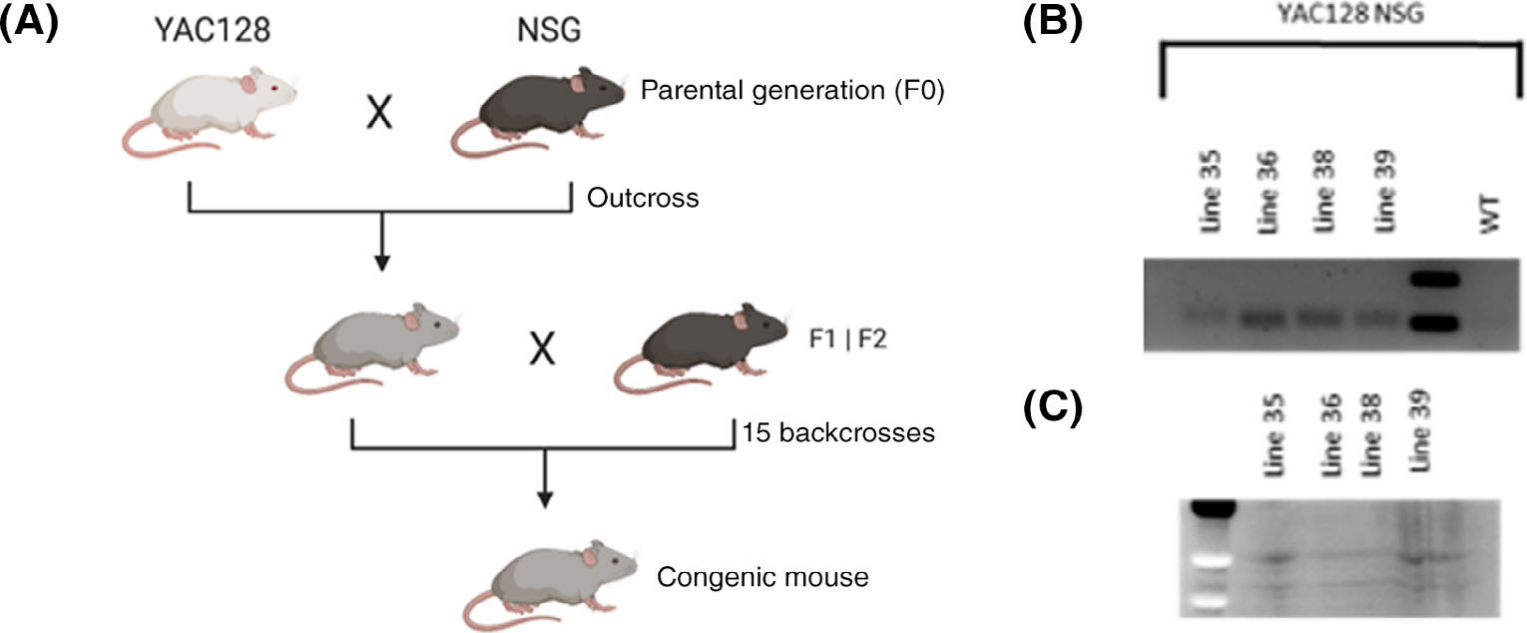
A, Creation of YACNSG line created by backcrossing the YAC128 (Slow et al^10^) mouse model with the immune deficient NSG background for 15 generations. B, RNA analysis from YACNSG mouse brains reveal expression of the htt transcript. C, Western blot analysis from the YACNSG brain reveal expression of expanded mutant human huntingtin

Four independent lines of YAC128‐NSG (YACNSG) were created by backcrossing the YAC128 mouse with an immune deficient NSG background for 15 generations (Figure 1A). The four mouse strains were genotyped for four primary NSG mutations for the first 10 generations in addition to the Htt expansion. RT‐PCR was performed from the brains of YACNSG and all lines demonstrated a band signifying that the mutant transcript was active following backcrossing on the NSG (Figure 1B). Protein was isolated from the four lines of YACNSG and an allele separation Western Blot was performed with MAB2166 (Millipore). All transgenic lines demonstrated the mutant and wildtype alleles at the protein level in the brain (Figure 1B).

**FIGURE 2:**
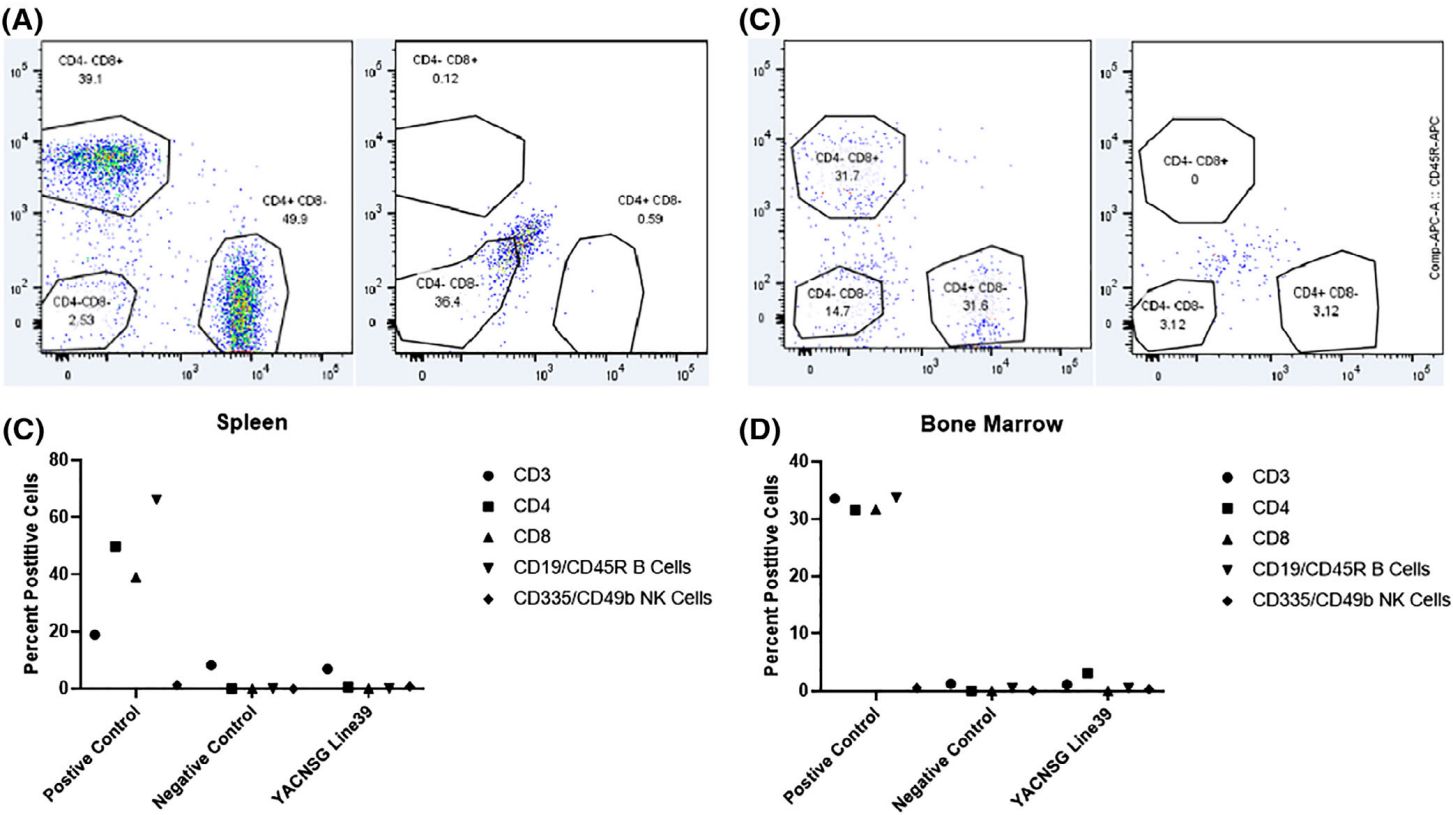
A, Representative flow cytometry from the spleen of positive control (left panel) and YACNSG line 39 (right panel) mice. B, Immune cell characterization from the spleen via flow cytometry. C, Representative flow cytometry from the bone marrow of positive control (left panel) and YACNSG line 39 (right panel) mice. D, Immune cell characterization from the bone marrow via flow cytometry

A comprehensive flow panel was performed from the blood, bone marrow, and spleen of the YACNSG strains to assess the presence of B cells, T cells, and NK cells. The four lines all demonstrated different levels of immune deficiency (Figure S1, Tables S1 and S2). YACNSG Line 38 demonstrated the highest level of immune deficiency in the Spleen (Figure 2A,B) and bone marrow (Figure 2C,D) and was chosen for more extensive characterization.

**FIGURE 3:**
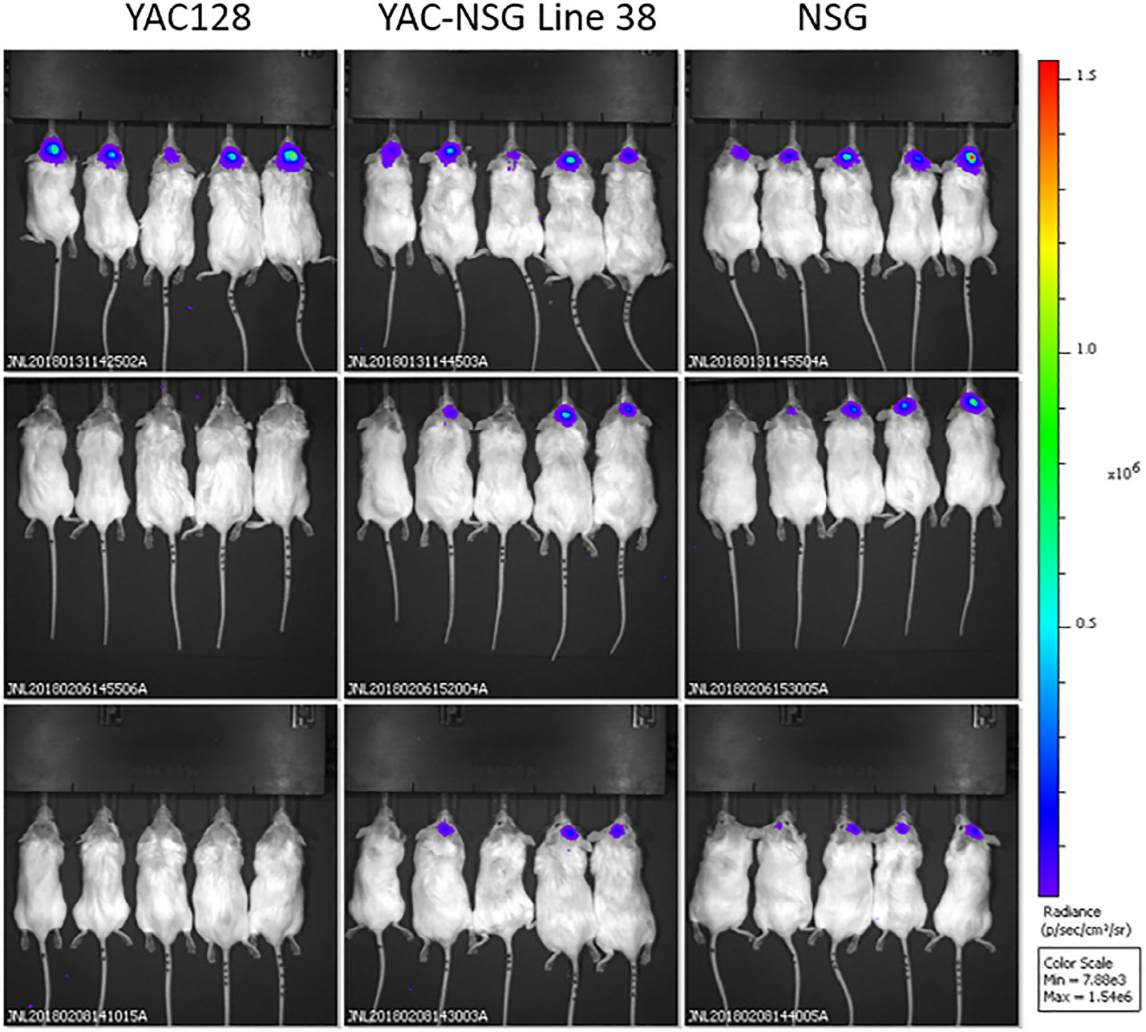
Xeno‐retention of transplanted human bone marrow derived MSC expressing luciferase. The novel immune deficient transgenic strains (YAC128/NSG) retained cells similar to the immune deficient NSG parental strain. Immune competent FVB/NJ mice rapidly cleared the transplanted cells. Taken together this data indicates that our novel strains are a viable option to test human derived cell products without the need for exogenous immune suppression

To further assess the level of immune deficiency and xenotolerance of the newly developed YACNSG strain, human MSC engineered with a lentivirus expressing green fluorescent protein (GFP) and luciferase were transplanted in the striatum following previously published methods (Pollock et al). Mice were imaged using bioluminescence imaging on an IVIS Spectrum (Perkin Elmer) starting 2 days following transplantation. In the parental immune competent FVB background of the YAC128 mouse, signal from the transplanted MSC was visible 48 hours after transplantation but had disappeared by 7 days. In the immune deficient background as well as in the novel YACNSG line, MSC were visible for at least 10 days suggesting that the newly developed line could tolerate xenotransplantation similar to NSG (Figure 3).

**FIGURE 4:**
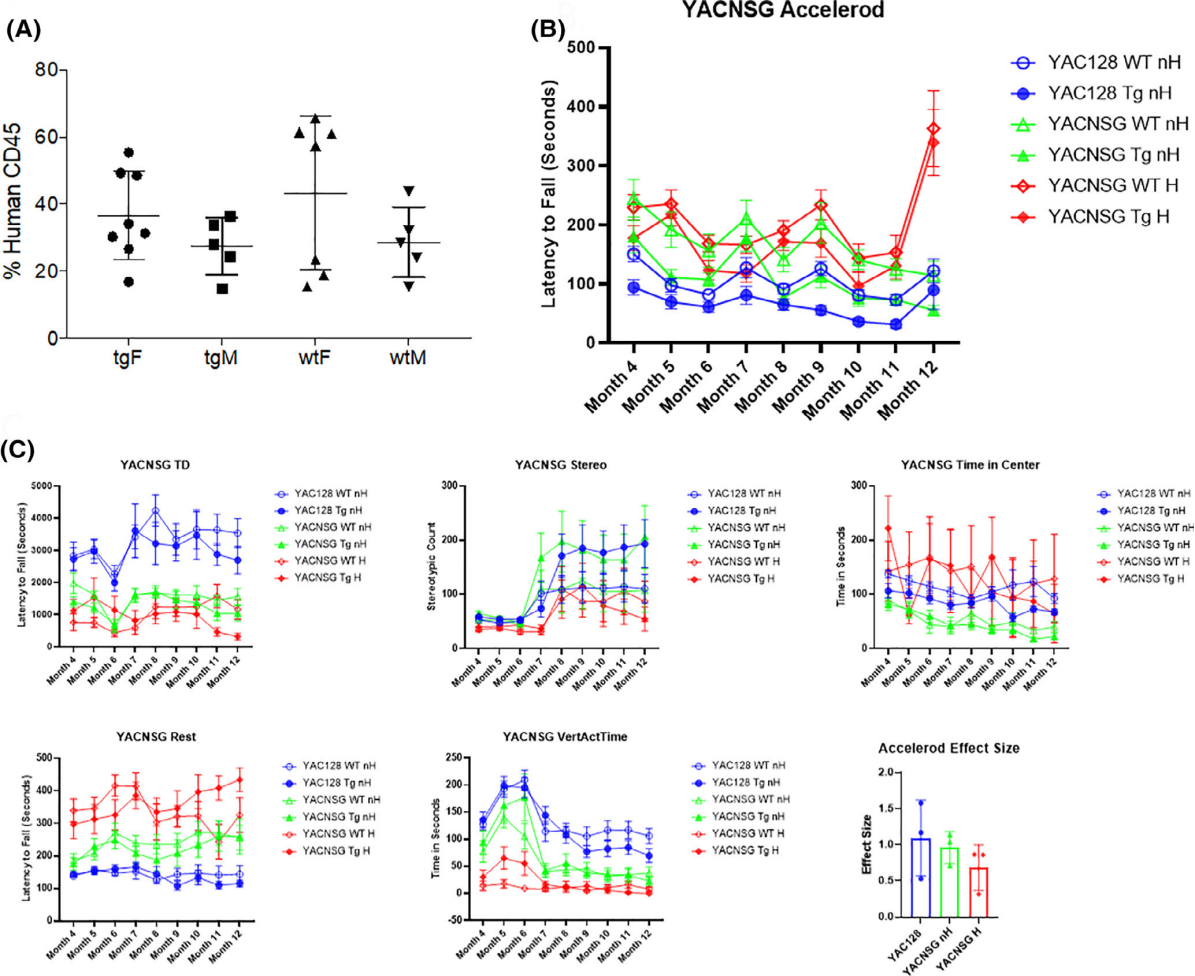
A, Human cell engraftment in the YAC128/NSG mouse strain. Male and female YAC128/NSG transgenic and wildtype littermates were sublethally irradiated and intrahepatically transplanted with cord blood derived CD34+ cells. Twelve weeks posttransplantation blood was collected via the tail vein and analyzed for human specific CD45 using flow cytometry. Mice selected for behavioral analysis displayed between 14.8% and 65.7% engraftment. No significant differences were observed between groups for level of engraftment. B, Accelerating rotarod was performed monthly for all groups. An overall repeated measures ANOVA interaction was observed. Post hoc revealed significant main effects between YAC128 WT and Tg mice as well as overall significance between the nonhumanized YACNSG WT and Tg. C, Open field assessment was performed for 10 minutes one time per month. Overall repeated measures ANOVA interactions were observed for total distance, rest time, and vertical activity. A within‐subject main effect was observed for stereotypic behavior and a between‐group effect was observed for time in the center quadrant. An analysis of effect size for the accelerod at 5‐, 9‐, and 11‐month time points revealed moderate to large effect size for each cohort of mice

To examine the impact of the immune system on the progression of HD and to extensively characterize the new strain with the parental YAC128 mouse, a longitudinal functional assessment was performed. A group of YACNSG were sub lethally irradiated at postnatal day 2‐5 and intrahepatically transplanted with human CD34+ derived from umbilical cord blood to provide the immune deficient mice with a human immune system.^25^ YACNSG were considered “humanized” if they demonstrated successful engraftment of greater than 25% human CD45+ at 3 months of age as measured by flow cytometry (Figure 4A). Functional assessment took place starting at 4 months of age and was performed monthly until the mice were 12 months of age. Functional assessment consisted of accelerod 4‐40 rpm and open field.

Accelerod analysis revealed an overall repeated measures ANOVA interaction *F*(40, 512) = 5.209, *P* < .001; and between group ANOVA *F*(5, 64) = 17.477, *P* < .001. LSD post hoc YAC128 WT nonhumanized (nH) significant to YAC128 Tg nH (0.05). The YACNSG WT nH significant to YACNSG Tg nH (0.005), nonsignificant to YACNSG WT humanized (H) (0.078) or YACNSG Tg H (0.436). YACNSG WT H nonsignificant to YACNSG Tg H (0.338). YACNSG Tg nH is significant to YACNSG Tg H (0.001) (Figure 4B). The effect size of each group (YAC128, YACNSG nH, YAC128 H) was assessed at 6‐, 9‐, and 11‐months of age. One‐way ANOVA revealed no significant between‐group differences *F*(2, 6) = 0.9309, *P* = .4445. The mean of the effect size across these timepoints suggest a medium and large effect size (0.67 and 0.95) for the novel YACNSG strain following humanization or nonhumanization, respectively compared with the parental YAC128 line (1.091). These results suggest that our novel YACNSG mouse strain has progressive motor impairment similar to the parental strain with humanization altering motor performance at specific disease phases.

Total distance analysis from the open field revealed overall repeated measures ANOVA interaction *F*(40, 512) = 1.557, *P* = .018; and between group ANOVA *F*(5, 64) = 6.216, *P* < .001. LSD post hoc YAC128 WT nH nonsignificant to YAC128 Tg nH (0.424), but significant to all YACNSG lines (*P* < .005) (Figure 4C). These results suggest that the total distance traveled by all NSG background strains were different from the parental YAC128 mice.

Stereotypic behavior analysis from the open field revealed overall repeated measures ANOVA within‐subject of time *F*(8, 512) = 15.840, *P* < .001. LSD post hoc no significant groups from the repeated measures ANOVA (Figure 4C).

Rest time analysis from the open field revealed repeated measures ANOVA interaction *F*(40, 512) = 2.305, *P* < .001; between group ANOVA *F*(5, 64) = 14.149, *P* < .001. LSD post hoc YAC128 WT nH nonsignificant to YAC128 Tg nH (0.424), but significant to all YACNSG lines (*P* < .005). YACNSG WT nH is nonsignificant to YACNSG Tg nH (0.595) or YACNSG WT H (0.059) but is significant to YACNSG Tg H (0.026). YACNSG Tg nH is significant to YACNSG Tg H (0.011) (Figure 4C). These results suggest that the novel YACNSG strain displays similar stereotypic behavior to the parental YAC128 mouse and that this may be a useful phenotypic measure.

Time in center analysis from the open field revealed repeated measures between group ANOVA *F*(5, 64) = 2.486, *P* = .04. LSD post hoc YAC128 WT nH nonsignificant to YAC128 Tg nH (0.424), but significant to all YACNSG nH lines (*P* < .005) (Figure 4C).

Vertical activity time analysis from the open field revealed repeated Measures ANOVA interaction *F*(40, 512) = 2.581, *P* < .001; between group ANOVA *F*(5, 64) = 14.876, *P* < .001. LSD post hoc YAC128 WT nH nonsignificant to YAC128 Tg nH (0.424), but significant to all YACNSG lines (*P* < .005). YACNSG WT nH significant to YACNSG WT H (0.025). YACNSG Tg nH significant to YACNSG Tg H (0.031) (Figure 4C). The vertical activity time results suggest an impact of the NSG background as well as the humanization protocol. While the novel YACNSG mice did not have phenotypic deficits at late stage of disease, trends toward significant differences were observed in early stages of disease progression.

**FIGURE 5:**
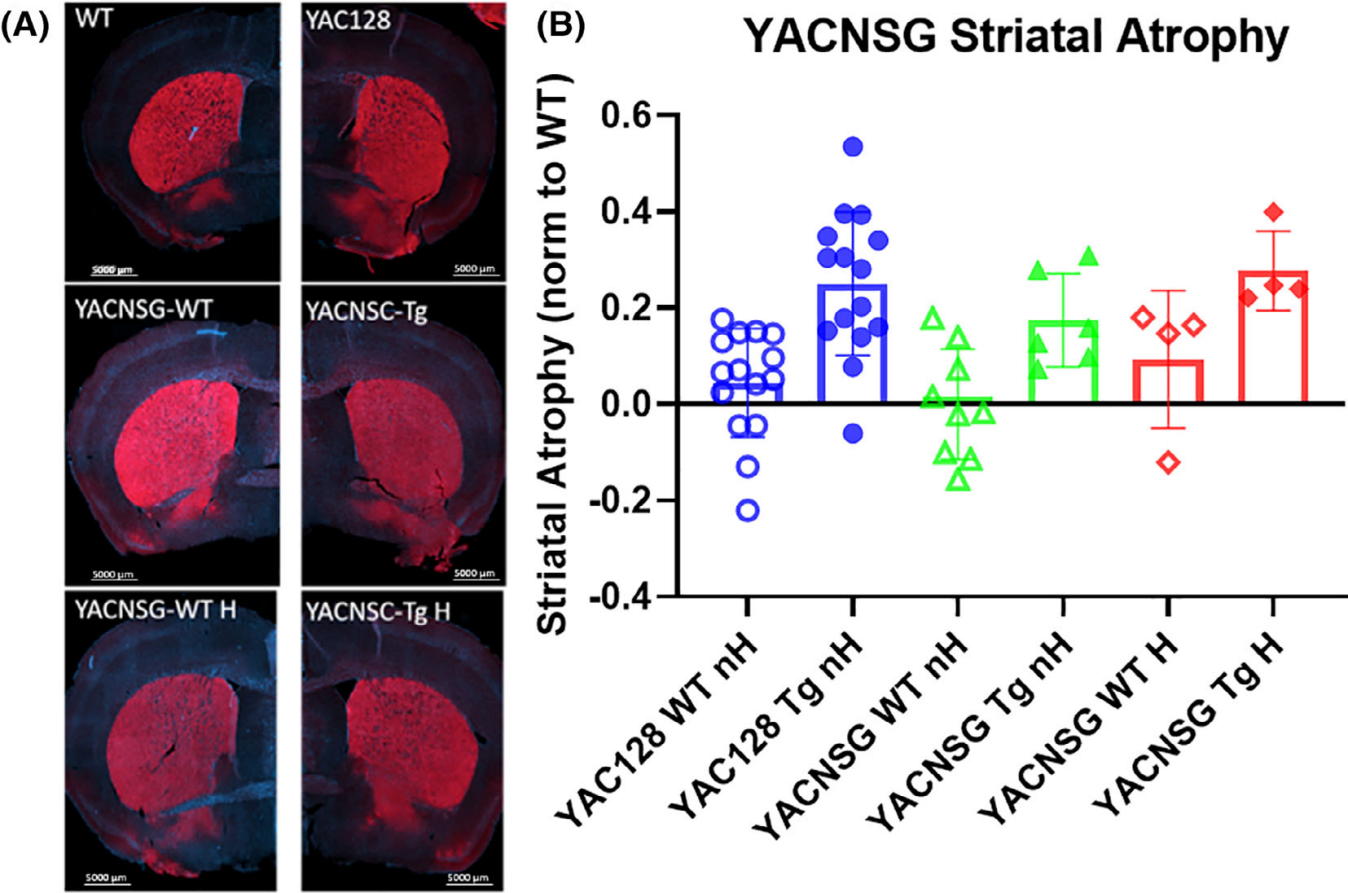
A, Striatal volume was assessed from coronal sections as previously described using a Darpp32 antibody. Slides were imaged using an AxioScan and volume was calculated using Zen Blue. Striatal volume was normalized to the wildtype littermate and atrophy was calculated as 1 − (volume/average volume of WT strain). B, An overall effect was observed with a one‐way ANOVA. Tukey's post hoc revealed a significant phenotype between the YAC128 WT and Tg; the YACNSG H WT and Tg, but not between the YACNSG nH WT and Tg. This data demonstrates the role of the immune cells in the neurodegenerative process of the YAC128 mouse model

At the conclusion of functional assessment, the mice were perfused and the brains were prepared for histology. Striatal volume was calculated following previously published protocols^26^ by measuring the area of Darpp32 (Figure 5A). Striatal volume analysis revealed one‐way ANOVA *F*(5, 53) = 5.885, *P* < .001. Tukey's post hoc revealed YAC128 WT nH were significant to YAC128 Tg nH (0.001) and YACNSG Tg H (0.033), nonsignificant to YACNSG Tg nH (0.210). All WT groups are nonsignificant to each other. YACNSG WT nH were significant from YAC128 Tg nH (0.006) and YACNSG Tg H (0.05) but nonsignificant from YACNSG Tg nH (Figure 5B). These results suggest that the novel YACNSG mice undergo striatal atrophy similar to the parental YAC128 mice. Interestingly, in the presence of a human immune system, the level of striatal atrophy more closely resembles that of the YAC128, suggesting a role for neuroinflammation on striatal degeneration.

## 5 DISCUSSION

The present study describes a novel animal model of Huntington's disease that allows for the careful study of the role of the human immune system on disease progression, and allows for the development of human cellular products to be tested in a disease background without the concern of rejection due to a xenotransplantation immune response. A novel line of YAC128 transgenic mice on an immune deficient NSG background strain was successfully generated, the YACNSG. This mouse model demonstrated the presence of the mutant transcript and protein in the brain in conjunction with a lack of circulating T, B, and natural killer cells.

The novel YACNSG transgenic mouse strain allows for the development of human cell transplantation products in a disease context.^27^, ^28^, ^29^, ^30^, ^31^, ^32^, ^33^ The results from this study suggest that adult human bone marrow derived mesenchymal stem (hMSC) cells engineered to express GFP and luciferase are retained longer than the YAC128 and similar to NSG mice. Our data demonstrates that cells are rapidly cleared from the CNS when using the parental YAC128 line without immune suppression as expected. Our previous work demonstrated that hMSC can survive for approximately 6 weeks when the mice are treated with a continuous administration via osmotic pumps of a combination of FK506 and rapamycin. While that approach does allow for the assessment of human cell efficacy over that time frame, the use of immune suppression is expensive, can result in abnormal functional efficacy,^34^, ^35^, ^36^ and limits any longitudinal assessment to the timeframe the mice are receiving the drug, which is usually only 4 to 6 weeks due to logistics of the alzet pump. However, the YAC128 mouse strain presents a great model to assess novel therapeutic development due to the progressive onset of motor, cognitive and neuropathological deficits.

The novel YACNSG mouse strain allows for the careful assessment of the role of the immune system on disease progression. To assess the role of the immune system, a cohort of age‐ and sex‐matched YACNSG mice were given a human immune system at postnatal day 2‐5 following previously established protocols.^23^, ^24^, ^25^ The humanized YACNSG were then longitudinally assessed alongside the parental YAC128 strain and nonhumanized YACNSG. Following assessment of the level of “humanization”, functional assessment began at 4 months and was continued until the mice were 12 months old. The YACNSG mice were able to demonstrate reconstitution of human immune cells in circulation similar to previous reports.^37^, ^38^ Analysis of the accelerod, a measure of motor coordination, revealed that the YAC128 and nonhumanized YACNSG mice demonstrated a reproducible phenotypic difference, as expected. While the Humanized YACNSG did not reach an overall repeated measures significant effect, many individual timepoints reveal a robust motoric phenotype. When assessing the effect size at three commonly used timepoints in the YAC128 (6‐, 9‐, and 11‐months) a medium to large effect size was observed, suggesting that measurements of motor phenotypes in this strain serves as a valuable outcome measure. Open field was also used to assess spontaneous exploration and overall motoric function. Multiple measures in the open field are suggestive of overall phenotypes between the WT and Tg from each respective cohort with interesting findings demonstrating the role of the immune system on disease progression.

At the conclusion of functional assessments, the brains of the YAC128 or YACNSG mice were prepared for histology following previously published protocols. The YAC128 displayed a typical 30% striatal atrophy when compared with their WT littermates. Interestingly, the YACNSG nonhumanized mice, while still showing significant striatal atrophy when compared with their WT littermates, were much reduced compared with the parental strain. The humanized YACNSG mice also displayed significant striatal atrophy when compared with their WT littermates, and the level of atrophy more closely resembled the parental YAC128 strain than the nonhumanized YACNSG. This data is suggestive that, at least for neuropathology, the immune system plays a large role in the loss of medium spiny neurons and striatal atrophy. This data would be supportive of the preclinical and clinical development of immune targeting molecules,^39^, ^40^, ^41^, ^42^ and while the SIGNAL and LEGATO‐HD trials have mixed clinical outcomes, targeting the neuroinflammation associated with disease onset and progression may result in clinically meaningful outcomes.

## 6 CONCLUSION

In summary, this study describes a novel mouse strain of Huntington's disease that allows for both the development of human cellular products for novel interventions and further describes the impact of the immune system on disease progression. Future studies will focus on characterizing the extent of humanization observed and the further study the role of neuroinflammation both in the presence and absence of cell or gene therapy.

## CONFLICT OF INTEREST

The authors declared no potential conflicts of interest.

## AUTHOR CONTRIBUTIONS

H.D., D.C., A.B., K.P., W.C., K.H.: conception and design, collection and/or assembly of data; S.Y., M.P., H.N., P.D.: collection and/or assembly of data; J.W.: conception and design; J.S.A.: conception and design, financial support, collection and/or assembly of data, data analysis and interpretation; K.F.: conception and design, financial support, collection and/or assembly of data, data analysis and interpretation, manuscript writing; J.N.: conception and design, financial support, administrative support, data analysis and interpretation, manuscript writing, final approval of manuscript.

## Supporting information


**Figure S1** Detailed plots of flow cytometry results from the blood and bone marrow of the YACNSG line. All samples are gated to an NSG background mouse.

**Table S1** Detailed summary of flow cytometry results in the YAC128/NSG lines. All strains normalized to NSG background mouse and gated from aqua viability and CD45+. Numbers reported are % over or under NSG control mouse.

**Table S2** Summary of all YAC128/NSG lines created at the UC Davis Stem Cell Program. All created mouse strains displayed mHTT at the RNA and protein level. A composite summary of T, B, and NK cells from the spleen, bone marrow, and blood displayed the immune deficient status of each line. Line 35 and 38 were selected for continued breeding and use in future experiments.

## Data Availability

The data that support the findings of this study are available from the corresponding author upon reasonable request.

## References

[1] Kremer B , Goldberg P , Andrew SE , et al. A worldwide study of the huntington's disease mutation: the sensitivity and specificity of measuring CAG repeats. N Engl J Med. 1994;330:1401‐1406. 8159192 10.1056/NEJM199405193302001

[2] Gusella JF , MacDonald ME . Huntington's disease and repeating trinucleotides. N Engl J Med. 1994;330:1450‐1451. 8159202 10.1056/NEJM199405193302011

[3] Liu L , Prime ME , Lee MR , et al. Imaging mutant Huntingtin aggregates: development of a potential PET ligand. J Med Chem. 2020;63:8608‐8633. 32662649 10.1021/acs.jmedchem.0c00955

[4] The Huntington's Collaborative Research Group . A novel gene containing a trinucleotide repeat that is expanded and unstable on Huntington's disease chromosomes. Cell. 1993;72:971‐983. 8458085 10.1016/0092-8674(93)90585-e

[5] Kopito RR . Aggresomes, inclusion bodies and protein aggregation. Trends Cell Biol. 2000;10:524‐530. 11121744 10.1016/s0962-8924(00)01852-3

[6] Gunawardena S , Her LS , Brusch RG , et al. Disruption of axonal transport by loss of huntingtin or expression of pathogenic polyQ proteins in Drosophila. Neuron. 2003;40:25‐40. 14527431 10.1016/s0896-6273(03)00594-4

[7] Crotti A , Glass CK . The choreography of neuroinflammation in Huntington's disease. Trends Immunol. 2015;36:364‐373. 26001312 10.1016/j.it.2015.04.007 PMC4786070

[8] Lois C , González I , Izquierdo‐García D , et al. Neuroinflammation in Huntington's disease: new insights with 11C‐PBR28 PET/MRI. ACS Chem Nerosci. 2018;9:2563‐2571. 10.1021/acschemneuro.8b00072 29719953

[9] Rocha NP , Ribeiro FM , Furr‐Stimming E , Teixeira AL . Neuroimmunology of Huntington's disease: revisiting evidence from human studies. Mediators Inflamm. 2016;2016:1‐10. 10.1155/2016/8653132 PMC4992798 27578922

[10] Slow EJ , van Raamsdonk J , Rogers D , et al. Selective striatal neuronal loss in a YAC128 mouse model of Huntington disease. Hum Mol Genet. 2003;12:1555‐1567. 12812983 10.1093/hmg/ddg169

[11] Pollock K , Stewart H , Cary W , et al. Mesenchymal stem cells engineered to produce BDNF for the treatment of Huntington's disease. Cell Transplant. 2014;23:780.

[12] Ballini A , Scacco S , Coletti D , Pluchino S , Tatullo M . Mesenchymal stem cells as promoters, enhancers, and playmakers of the translational regenerative medicine. Stem Cells Int. 2017;2017:1‐2. 10.1155/2017/3292810 PMC5504961 28740512

[13] Ballini A , Cantore S , Scacco S , et al. Mesenchymal stem cells as promoters, enhancers, and playmakers of the translational regenerative medicine 2018. Stem Cells Int. 2018;2018:2018. 10.1155/2018/6927401 PMC6232791 30510586

[14] Bechstein WO . Neurotoxicity of calcineurin inhibitors: impact and clinical management. Transpl Int. 2000;13:313‐326. 11052266 10.1007/s001470050708

[15] Kawakami M , Yoshimoto T , Nakagata N , Yamamura KI , Siesjo BK . Effects of cyclosporin a administration on gene expression in rat brain. Brain Inj. 2011;25:614‐623. 21534739 10.3109/02699052.2011.571229

[16] Gijtenbeek JM , van den Bent MJ , Vecht CJ . Cyclosporine neurotoxicity: a review. J Neurol. 1999;246:339‐346. 10399863 10.1007/s004150050360

[17] De Mattos AM , Olyaei AJ , Bennett WM . Nephrotoxicity of immunosuppressive drugs: long‐term consequences and challenges for the future. Am J Kidney Dis. 2000;35:333‐346. 10676738 10.1016/s0272-6386(00)70348-9

[18] Coughlan AM , Harmon C , Whelan S , et al. Myeloid engraftment in humanized mice: impact of granulocyte‐colony stimulating factor treatment and transgenic mouse strain. Stem Cells Dev. 2016;25:530‐541. 26879149 10.1089/scd.2015.0289

[19] Shultz LD , Lyons BL , Burzenski LM , et al. Human lymphoid and myeloid cell development in NOD/LtSz‐ scid IL2R γ null mice engrafted with mobilized human Hemopoietic stem cells. J Immunol. 2005;174:6477‐6489. 15879151 10.4049/jimmunol.174.10.6477

[20] Pollock K , Dahlenburg H , Nelson H , et al. Human mesenchymal stem cells genetically engineered to overexpress brain‐derived neurotrophic factor improve outcomes in Huntington's disease mouse models. Mol Ther. 2016;24:965‐977. 26765769 10.1038/mt.2016.12 PMC4881765

[21] Fierro FA , O'Neal AJ , Beegle JR , et al. Hypoxic pre‐conditioning increases the infiltration of endothelial cells into scaffolds for dermal regeneration pre‐seeded with mesenchymal stem cells. Front Cell Dev Biol. 2015;3:1‐9. 26579521 10.3389/fcell.2015.00068 PMC4626656

[22] Beegle J , Lakatos K , Kalomoiris S , et al. Hypoxic preconditioning of mesenchymal stromal cells induces metabolic changes, enhances survival, and promotes cell retention in vivo. Stem Cells. 2015;33:1818‐1828. 25702874 10.1002/stem.1976 PMC10757456

[23] Beegle J , Hendrix K , Maciel H , Nolta JA , Anderson JS . Improvement of motor and behavioral activity in Sandhoff mice transplanted with human CD34+ cells transduced with a HexA/HexB expressing lentiviral vector. J Gene Med. 2020;22:e3205. 32335981 10.1002/jgm.3205 PMC12067969

[24] Barclay SL , Yang Y , Zhang S , et al. Safety and efficacy of a tCD25 preselective combination anti‐HIV lentiviral vector in human hematopoietic stem and progenitor cells. Stem Cells. 2015;33:870‐879. 25524029 10.1002/stem.1919

[25] Walker JE , Chen RX , McGee J , et al. Generation of an HIV‐1‐resistant immune system with CD34+ hematopoietic stem cells transduced with a triple‐combination anti‐HIV Lentiviral vector. J Virol. 2012;86:5719‐5729. 22398281 10.1128/JVI.06300-11 PMC3347262

[26] Pollock K , Dahlenburg H , Nelson H , et al. Human mesenchymal stem cells genetically engineered to overexpress brain‐derived neurotrophic factor improve outcomes in Huntington's disease mouse models. Mol Ther. 2016;24:965‐977. 26765769 10.1038/mt.2016.12 PMC4881765

[27] Ebrahimi T , Abasi M , Seifar F , et al. Transplantation of stem cells as a potential therapeutic strategy in neurodegenerative disorders. Curr Stem Cell Res Ther. 2020;15:133‐144. 10.2174/1574888X15666200628141314 32598273

[28] Wang J , Hu WW , Jiang Z , Feng MJ . Advances in treatment of neurodegenerative diseases: perspectives for combination of stem cells with neurotrophic factors. World J Stem Cells. 2020;12:323‐338. 32547681 10.4252/wjsc.v12.i5.323 PMC7280867

[29] Björklund A , Parmar M . Neuronal replacement as a tool for basal ganglia circuitry repair: 40years in perspective. Front Cell Neurosci. 2020;14:1‐22. 32547369 10.3389/fncel.2020.00146 PMC7272540

[30] Kim HS , Jeon I , Noh J‐E , et al. Intracerebral transplantation of BDNF‐overexpressing human neural stem cells (HB1.F3.BDNF) promotes migration, differentiation and functional recovery in a rodent model of Huntington's disease. Exp Neurobiol. 2020;29:130‐137. 32408403 10.5607/en20011 PMC7237270

[31] Yoon Y , Kim HS , Jeon I , et al. Implantation of the clinical‐grade human neural stem cell line, CTX0E03, rescues the behavioral and pathological deficits in the quinolinic acid‐lesioned rodent model of Huntington's disease. Stem Cells. 2020;38:936‐947. 32374064 10.1002/stem.3191 PMC7496241

[32] Besusso D , Schellino R , Boido M , et al. Stem cell‐derived human striatal progenitors innervate striatal targets and alleviate sensorimotor deficit in a rat model of Huntington disease. Stem Cell Rep. 2020;14:876‐891. 10.1016/j.stemcr.2020.03.018 PMC7220987 32302555

[33] Reidling JC , Relaño‐Ginés A , Holley SM , et al. Human neural stem cell transplantation rescues functional deficits in R6/2 and Q140 Huntington's disease mice. Stem Cell Rep. 2018;10:58‐72. 10.1016/j.stemcr.2017.11.005 PMC5768890 29233555

[34] Rosenstock TR , De Brito OM , Lombardi V , et al. FK506 ameliorates cell death features in Huntington's disease striatal cell models. Neurochem Int. 2011;59:600‐609. 21703318 10.1016/j.neuint.2011.04.009

[35] Kumar P , Kumar A . Neuroprotective effect of cyclosporine and FK506 against 3‐nitropropionic acid induced cognitive dysfunction and glutathione redox in rat: possible role of nitric oxide. Neurosci Res. 2009;63:302‐314. 19367792 10.1016/j.neures.2009.01.005

[36] Kumar P , Kalonia H , Kumar A . Possible nitric oxide modulation in protective effect of FK‐506 against 3‐nitropropionic acid‐induced behavioral, oxidative, neurochemical, and mitochondrial alterations in rat brain. Drug Chem Toxicol. 2010;33:377‐392. 20550427 10.3109/01480541003642050

[37] Anselmi G , Helft J , Guermonprez P . Development and function of human dendritic cells in humanized mice models. Mol Immunol. 2020;125:151‐161. 32688117 10.1016/j.molimm.2020.07.005

[38] Walcher L , Hilger N , Wege AK , et al. Humanized mouse model: hematopoietic stemcell transplantation and tracking using short tandem repeat technology. Immun Inflamm Dis. 2020;8:363‐370. 32525618 10.1002/iid3.317 PMC7416029

[39] Di Pardo A , Ciaglia E , Cattaneo M , et al. The longevity‐associated variant of BPIFB4 improves a CXCR4‐mediated striatum‐microglia crosstalk preventing disease progression in a mouse model of Huntington's disease. Cell Death Dis. 2020;11:546. 32683420 10.1038/s41419-020-02754-w PMC7368858

[40] Fatoba O , Ohtake Y , Itokazu T , Yamashita T . Immunotherapies in Huntington's disease and α‐synucleinopathies. Front Immunol. 2020;11:1‐17. 32161599 10.3389/fimmu.2020.00337 PMC7052383

[41] Björkqvist M , Wild EJ , Thiele J , et al. A novel pathogenic pathway of immune activation detectable before clinical onset in Huntington's disease. J Exp Med. 2008;205:1869‐1877. 18625748 10.1084/jem.20080178 PMC2525598

[42] Denis HL , Lauruol F , Cicchetti F . Are immunotherapies for Huntington's disease a realistic option? Mol Psychiatry. 2019;24:364‐377. 29487401 10.1038/s41380-018-0021-9

