# Supplementary figures and images for: A novel Huntington's disease mouse model to assess the role of neuroinflammation on disease progression and to develop human cell therapies
Source: Stem Cells Transl Med. 2021 Mar 12;10(7):1033–43. doi: 10.1002/sctm.20-0431 (PMC8235129; doi:10.1002/sctm.20-0431)

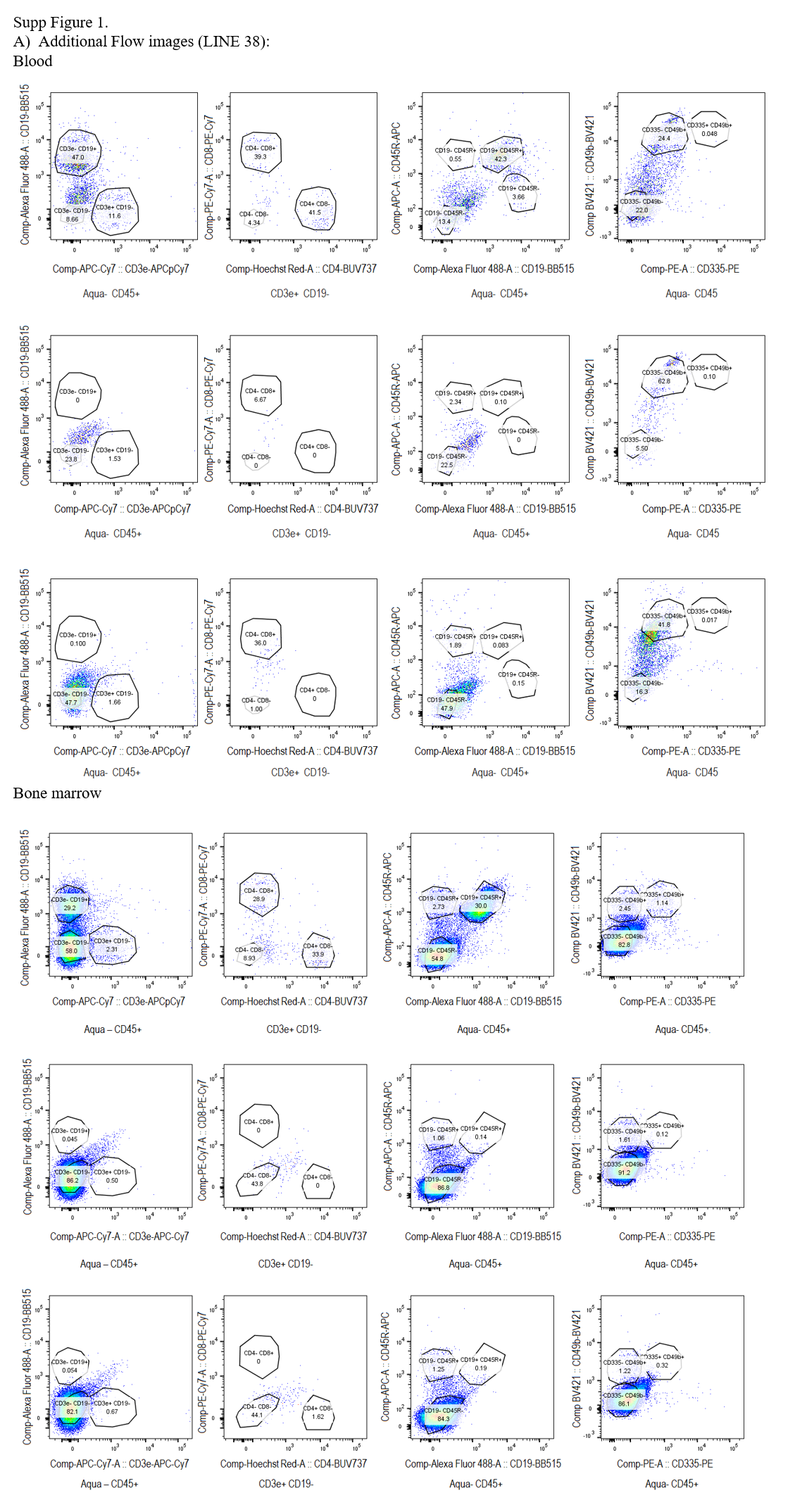

Supplement: Supplementary file 1 — Figure S1 Detailed plots of flow cytometry results from the blood and bone marrow of the YACNSG line. All samples are gated to an NSG background mouse. [file SCT3-10-1033-s002.tif]

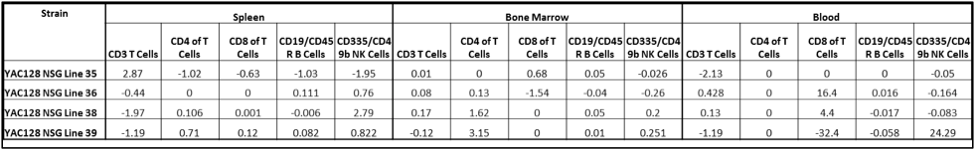

Supplement: Supplementary file 2 — Table S1 Detailed summary of flow cytometry results in the YAC128/NSG lines. All strains normalized to NSG background mouse and gated from aqua viability and CD45+. Numbers reported are % over or under NSG control mouse. [file SCT3-10-1033-s003.tif]

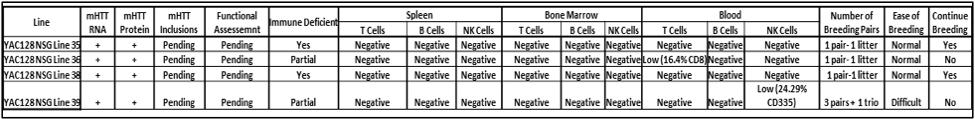

Supplement: Supplementary file 3 — Table S2 Summary of all YAC128/NSG lines created at the UC Davis Stem Cell Program. All created mouse strains displayed mHTT at the RNA and protein level. A composite summary of T, B, and NK cells from the spleen, bone marrow, and blood displayed the immune deficient status of each line. Line 35 and 38 were selected for continued breeding and use in future experiments. [file SCT3-10-1033-s001.tif]
